# Successful Treatment with Thrombopoietin Receptor Agonist in Avoiding Splenectomy for Patients with Chronic Refractory Immune Thrombocytopenia

**DOI:** 10.4084/MJHID.2012.003

**Published:** 2012-01-18

**Authors:** Alhossain Khalafallah, Zafreen Rahman, Kath Ogden, Terry Hannan

**Affiliations:** 1 Launceston General Hospital, University of Tasmania, Australia; 2 School of Human Life Sciences, University of Tasmania, Australia; 3 Launceston Clinical School, University of Tasmania, Australia

## Abstract

**Background:**

Chronic immune thrombocytopenia (ITP) is a condition associated with significant morbidity; however the management options are often unsatisfactory with a portion of patients exhibiting a refractory-relapsing disease path despite various lines of treatment including splenectomy. As a thrombopoietin receptor agonist, eltrombopag (GlaxoSmithKline, Australia) provides a novel treatment option for patients with refractory disease. We describe the outcomes of four patients with chronic ITP, who were treated with eltrombopag as a single agent.

**Methods:**

Four Caucasian patients with chronic refractory ITP (2 males; 2 females) were enrolled in this study with a mean age of 48 years (range, 39–59). All patients were non-splenectomised and were refractory to several lines of treatment including steroids, intravenous immunoglobulin, vincristine, and azathioprine, one patient has also received rituximab (a monoclonal antibody that binds the CD20 antigen expressed by B-lymphocytes). All patients were treated with oral eltrombopag (50–75 mg) for a median period of 12 months (range, 9–16).

**Results:**

After a median follow up of 20 months (range, 11–34), platelet counts recovered to normal levels in two patients. One recovered a normal platelet count after 13 months, the other 34 months of completion of treatment with eltrombopag. No additional immune suppressive therapy was required.

The other two patients also discontinued eltrombopag at 27 and 11 months after achievement of satisfactory platelet counts above 30/nL without any bleeding complications. Other forms of immune therapy were also ceased in these two cases. None of the four patients required splenectomy.

**Conclusion:**

The clinical outcomes in this small cohort of patients suggests that eltrombopag may have a role to play in the long term control of chronic ITP whilst avoiding splenectomy and long term immunosuppressive therapy. The beneficial outcomes in our patients led to a sustained elevation in platelets with no adverse effects noted when used for relatively longer periods than previously reported. It is worth noting that spontaneous remission does occur with ITP and is the most likely cause for the favourable outcome with eltrombopag therapy. However, if eltrombopag is able to reduce the need for splenectomy in patients with chronic ITP then a distinct quality of care outcome can be achieved by avoiding the recognised short- and long-term complications of splenectomy. Randomised controlled trials with long-term follow up are warranted.

## Introduction

Immune thrombocytopenia (ITP) is an autoimmune condition mediated by the production of autoantibodies directed against platelets.^1^–^3^ Thus in ITP there is a premature removal of platelets by the cells of the reticulo-endothelial system, accelerated platelet destruction and to some extent impaired platelet production.^1^,^2^ The clinical presentation is dependent on the degree of thrombocytopenia. Minor symptoms include easy bruising and epistaxis, however when the platelet count drops to below 10/nL, severe spontaneous bleeding such as an intracranial hemorrhage can occur.^1^–^3^

The primary aim of ITP treatment is to maintain a stable platelet count at a level that prevents bleeding events^3^–^5^ without applying the adverse effects of immunosuppressive medical therapies or surgical intervention.

Typically, therapies for ITP have focused on preventing platelet destruction using glucocorticoids, immunoglobulins, immunosuppressive agents and splenectomy. However, these therapies have been unsuccessful up to 30–40% of patients, particularly in chronic refractory ITP.^3^–^5^ It has also been noted that platelet production is often suboptimal in ITP. Therefore novel therapies with thrombopoietic receptor agonists such as oral eltrombopag^6^–^9^ and subcutaneous romiplostim^10^–^12^ are focusing on enhancing platelet production and have been explored as an alternative to immunosuppressive therapy.

Eltrombopag is an oral small molecule, non-peptide thrombopoietin (TPO) receptor agonist which binds to the transmembrane domain of the TPO receptor and induces the proliferation of cells within the megakaryocyte lineage.^13^ As eltrombopag and TPO do not bind to the same site on the TPO receptor, and there is no competitive binding, therefore eltrombopag and TPO are able to act in conjunction with each other, resulting in an additive cell-signalling effect that has shown efficacy and safety in patients with chronic ITP.^6^–^9^

Various clinical studies have been carried out with this novel treatment. Phase I clinical trials were first conducted in 2007, reporting an increase in platelet count after the administration of eltrombopag for ITP, with no adverse reactions occurring. Furthermore, platelet function was not affected by eltrombopag.^13^ A phase I/II study conducted in 2007 with 118 patients with refractory and chronic relapsing ITP demonstrated that most patients were able to maintain an elevated platelet count at higher doses of eltrombopag, and showed a trend towards fewer episodes of bleeding.^12^ Again, similar positive results were yielded from a multinational phase III randomised, double-blind, placebo-controlled trial of patients with chronic ITP.^11^,^14^

Following the successful introduction of TPO receptor agonists in trials where it was used as a second line treatment using different guidelines,^3^–^5^ this novel therapy proved promising for the future of patients suffering from chronic refractory ITP. It demonstrated the potential of avoiding poorly tolerated steroids, immunosuppressive therapies and invasive surgical interventions such as a splenectomy. If these therapeutic benefits are confirmed, we consider it is possible that TPO receptor agonists may one day become the first line treatment for chronic refractory ITP.

## Methods and Results

We describe four patients with the diagnosis of chronic ITP classified according to the international consensus report on the investigation and management of ITP^3^ and who presented to the Launceston General Hospital (LGH) between January 2008 and May 2010. Approval of the Human Ethics Committee of Tasmania, Australia was gained to conduct this study.

All patients were Caucasian, over 18 years of age with a mean age of 48 years (range, 39–59). There were two males and two females and all had been diagnosed with chronic refractory ITP for more than 6 months (Table 1) and were experiencing ITP associated bleeding.

Each patient had a normal bone marrow aspirate at the time of diagnosis, showing normal or active megakaryopoeisis as reviewed by the Hematologist and being consistent with ITP. No evidence of other hematological disorders such as lymphoma or Evans’ syndrome. None of these patients had evidence of infection with Hepatitis B or C virus, or Human Immunodeficiency Virus. Furthermore, a Helicobacter pylori infection was excluded in all subjects.^3^–^5^

The diagnosis of chronic refractory ITP was made in the setting of several clinical relapses in all patients due to inadequate control of the ITP by previous treatment methods.^3^ Each patient had received previous treatment for the ITP with glucocorticoids, intravenous immunoglobulin (IVIG) or immune modulators and one failed treatment with rituximab.

Patients who were concomitantly treated with steroid therapy had their steroid dose tapered down to a complete stop whilst on the eltrombopag treatment. None of the patients proceeded with splenectomy as they refused the procedure and opted for new therapy in order to control their difficult ITP.

The therapeutic regimen in all patients was a once daily dose of 50 or 75 mg of eltrombopag administered orally. The treatment period varied between 9 to 16 months (median, 12) and patients were followed up for a median period of 20 months (range, 11–34) (Table 1).

The patients platelet counts were measured regularly mostly on a weekly basis for all patients who were receiving different treatment lines. In addition, patient tolerability, drug safety, signs of bleeding, quality of life and any complications were recorded.

In two of the four patients, the platelet counts recovered to normal levels for at least 1 year after completion of treatment with eltrombopag. No further immune suppressive therapy was required and these patients currently have normal platelet counts at 34 and 13 months post eltrombopag-treatment.

The other two patients also had their eltrombopag discontinued after achievement of platelet counts between 50–100/nL without any bleeding complications. In the meantime, other forms of immune therapy were ceased. No patient proceeded to splenectomy as part of management of their refractory ITP (Table 1).

Within the study period of our cohort of patients in this report, we had investigated 47 patients with ITP at our hospital that were referred by either the General Practitioners (GP) or Emergency Department and were managed as the first line of treatment with steroids or IVIG therapy. Of these thirty patients (64%) responded to the first line treatment (with corticosteroids and/or IVIG therapy) and maintained their platelets at a satisfactory level with a platelet count above 30/nL, while 9 patients (19%) required additional courses of treatment including IVIG with a good response and maintained the platelet count above 30/nL.

Eight patients (17%) developed refractory ITP as defined by the International Consensus Report criteria^3^ and underwent a second line treatment, in which four (4) patients underwent splenectomy. The four (4) non-splenectomised patients were included in this current study and were treated with oral TPO-receptor agonist (Eltrombopag) available at the LGH as a part of the patient familiarization program for compassionate use only. Two patients who underwent splenectomy have recovered from ITP, while the other two patients required further treatment with the newly available TPO agonist, romiplostim (Nplate®, Amgen Australia) and have successfully maintained a satisfactory level of platelets without other forms of treatment for ITP.

## Discussion

Chronic refractory ITP is understood to be an autoimmune disorder.^1^–^5^ Therapeutic options that are often successful in patients with acute ITP such as corticosteroids, IVIG, alkylating agents and splenectomy, have a variable effect on chronic refractory ITP.^3^–^5^

Splenectomy is currently employed as a second line therapy, however it is ineffective in about 30–40% of the cases.^15^,^16^ Also these therapies present an increasing risk for opportunistic infection and post surgical complications during their treatment courses and adversely affect the overall outcomes.^16^

The human monoclonal antibody, rituximab, is a chimeric, IgG1/κ that is specific for the CD 20 antigen, expressed on the surface of B lymphocytes and employed in immune-mediated disorders.^17^ Furthermore, complement-dependent cytotoxicity is probably one of the mechanisms of action of rituximab and has been proposed by some authors as the most important mechanism in controlling different immune disorders.^17^ However, the success rate of rituximab in chronic refractory ITP remains variable, being around 20–40%.^3^,^18^,^19^ Furthermore in our study, patient 1 (Table 1) received, in addition to three different immunotherapies, rituximab without any response. Eltrombopag therapy was well tolerated without side effects. The platelet counts remain currently normal in two patients and above 30/nl in the other two despite discontinuation of the drug for more than 6 months and absence of other treatments.

In a literature review we found that splenectomy complications were reported around 12.9% with laparotomy and 9.6% with laparoscopy, with mortality rates of 1.0% with laparotomy and 0.2% with laparoscopy.^20^–^22^ With TPO-receptor agonists in patients after splenectomy there is approximately an 80% overall response rate.^3^–^5^

The International Consensus Report on ITP published by Provan and colleagues,^3^ defined first line treatment with corticosteroids and/or IVIG therapy and anti-D. All patients in our series had received the former treatments while the latter treatment (Anti-D) is not available in Australia.

The second line was referred to surgical intervention (splenectomy) or medical management consisting mainly of chemotherapeutic agents (e.g. vincristine, cyclophosphamide) including immunomodulators (e.g. rituximab, cyclosporin A) in addition to the novel agents with TPO-receptor agonists. The same expert review suggested that approximately 20% of patients do not attain a hemostatic platelet count after splenectomy with an additional 20% of splenectomised patients eventually relapsing.^3^

The American Society of Hematology (ASH) in 2011 published evidence-based practice guidelines for ITP and recommended TPO receptor agonists for patients at risk of bleeding who relapsed after splenectomy or had a contraindication to splenectomy.

At the same time the expert-consensus for the standardization of terminology, definitions and outcome criteria in ITP published by the International Working Group^5^ reported that refractory ITP patients should meet the criteria of a failed splenectomy and exhibit severe ITP complications. Although our cohort of patients did not undergo splenectomy, they have had severe ITP and failed first and second lines of medical treatment.

The ITP patients are predominantly managed by General Practitioners (GP) in Australia. However, they are referred to the Hematologist when the platelet count becomes very low, or when a bleeding complication occurs, or prior to surgical procedure for an expert opinion. Therefore, the 47 referred cases with ITP to the LGH during the study period does not reflect necessarily all cases of ITP diagnosed at the same time. It is beyond the scope of this study to analyse all cases of ITP that have been referred to the same institution.

## Conclusion

In our case-series, we believe that eltrombopag played a significant role in these non-splenectomised patients with chronic ITP. The results recorded here provide a further evidence for the long-term control of chronic ITP without requiring splenectomy. We also note that the newer thrombopoietic agents do not generally cure patients with ITP. From our results, even though the patient subgroup is small, we believe that the achievement of treatment independency with eltrombopag may be related to the natural history of ITP rather than the long-term effect of the TPO-receptor agonist.^3^ The maintenance of platelet-count after stopping eltrombopag could be attributed to a spontaneous remission that is not uncommon in ITP.^1^–^5^ The new TPO-receptor agonists may be considered an effective tool for obtaining safe levels of platelets waiting for a possible spontaneous remission of immune mechanisms that lead to ITP.^3^–^5^

Our results may indicate that patients with chronic and refractory ITP should be considered for treatment with thrombopoietin receptor agonists before proceeding with an irreversible invasive surgical procedure such as splenectomy. Employing thrombopoietin receptor agonists in this cohort of patients may avoid the known risks associated with splenectomy including general anesthesia risks, risk of complications during and after the procedure as well as increasing susceptibility for long-term infections.^20^–^23^ Further randomised trials to address this issue are warranted.

## Figures and Tables

**Table 1:** Patient demographic and characteristics.

	Patient 1	Patient 2	Patient 3	Patient 4

**Gender**	Male	Male	Female	Female
**Race**	Caucasian	Caucasian	Caucasian	Caucasian
**Diagnosis**	Chronic ITP	Chronic ITP	Chronic ITP	Chronic ITP
**Age at** diagnosis **(years)**	39	59	57	39
**Bone Marrow at diagnosis**	Normal	Normal	Normal	Normal
**Platelet count at diagnosis (×10^9^/L)**	1	12	10	<10
**lowest platelets count**	1	12	5	6
**Previous treatments**	High dose Prednisolone/high-dose Dexamethasone, IVIG Vincristine, Rituximab	Moderate dose Prednisolone 50–100mg, Imuran Vincristine	IVIG, Prednisolone moderate dose, high-dose Dexamethasone, Vincristine, Imuran	IVIG, Prednisolone moderate dose, high-dose Dexamethasone
**Treatment period in months**	9	16	11	12
**Dose (in mg)**	50–75	50	50	50–75
**Follow up post Eltrombopag (months)**	34	27	13	11
**Splenectomy**	None	None	None	None

Prednisolone moderate dose is between 25mg–75 mg, high-dose; between 500 mg to 1000mg. High-dose dexamethasone 40 mg. IVIG; intravenous immunoglobulin at dose of 1g/Kg body weight.
